# RETRACTED: De Vecchis et al. An Admission-to-Discharge BNP Increase Is a Predictor of Six-Month All-Cause Death in ADHF Patients: Inferences from Multivariate Analysis Including Admission BNP and Various Clinical Measures of Congestion. *J. Clin. Med.* 2016, *5*, 99

**DOI:** 10.3390/jcm13061661

**Published:** 2024-03-14

**Authors:** Renato De Vecchis, Carmelina Ariano, Cesare Baldi

**Affiliations:** 1 Cardiology Unit, Presidio Sanitario Intermedio “Elena d’Aosta”, ASL Napoli 1 Centro, via Cagnazzi 29, 80137 Napoli, Italy; carmelariano@tiscali.it; 2 Division of Cardiology, Casa di Cura “Sollievo della Sofferenza”, viale Cappuccini 2, 71013 San Giovanni Rotondo, Italy; 3 Heart Department, Interventional Cardiology, Azienda Ospedaliero-Universitaria “San Giovanni di Dio e Ruggi d’Aragona”, via San Leonardo 1, 84131 Salerno, Italy; cesare.baldi@tiscali.it

The *Journal of Clinical Medicine* retracts the article “An Admission-to-Discharge BNP Increase Is a Predictor of Six-Month All-Cause Death in ADHF Patients: Inferences from Multivariate Analysis Including Admission BNP and Various Clinical Measures of Congestion“ [1], cited above.

Following publication, concerns were brought to the attention of the publisher regarding potential redundancy concerning a previous publication [2] from the same authorship group.

Adhering to our complaint’s procedure, an investigation was conducted by the Editorial Office and the Editorial board that confirmed a significant overlap between the two publications. As a result, the Editorial Office and Editorial Board consider this publication [1] to be redundant and, therefore, have decided to retract, as per MDPI’s retraction policy (https://www.mdpi.com/ethics#_bookmark30), and in line with the Committee on Publication Ethics’ retraction guidelines (https://publicationethics.org/retraction-guidelines).

This retraction was approved by the Editor-in-Chief of the *Journal of Clinical Medicine*.

The authors did not provide a comment on this retraction decision.
